# Determinants of adherence to anti-TB treatment and associated factors among adult TB patients in Gondar city administration, Northwest, Ethiopia: based on health belief model perspective

**DOI:** 10.1186/s41043-021-00275-6

**Published:** 2021-11-27

**Authors:** Resom Berhe Gebremariam, Maereg Wolde, Aykerem Beyene

**Affiliations:** 1 Department of Health Education and Behavioral Sciences, Institute of Public Health, College of Medicine and Health Science, University of Gondar, P.O. Box 196, Gondar, Ethiopia; 2 Gondar Referral Hospital, Gondar, Ethiopia

**Keywords:** Treatment adherence, Health belief model, Tuberculosis treatment

## Abstract

**Background:**

Adherence is crucial in treating Tuberculosis to achieve the required treatment success rate. However, due to the longer treatment duration, adherence to Tuberculosis treatment is the most challenging factor affecting Tuberculosis control. Furthermore, although several studies have reported the determinants of Tuberculosis treatment adherence, few of them have used Health Belief Model (HBM) as the guiding principle to determine the individual perception of health-related decisions as much or more than medical concerns with Tuberculosis treatment adherence. Therefore, this study aims to assess adherence to anti-Tuberculosis treatment and associated factors among adult Tuberculosis patients in Gondar city, Northwest Ethiopia, in 2020.

**Methods:**

Institution-based cross-sectional study was conducted among Tuberculosis patients following anti-Tuberculosis treatment in Gondar city health facilities from February 20 to March 26, 2020. A total of 265 Tuberculosis patients were selected by systematic random sampling techniques that include patients who were on treatment follow up for ≥ 1 month and whose age is ≥ 18 years. Data were collected by trained data collectors using interviewer administer and structured questionnaires. EPI DATA version 4.2 was used for data entry and SPSS version 24 for analysis. The logistic regression model was used to indicate the association between independent variables with adherence to anti Tuberculosis treatment.

**Results:**

The overall rate of adherence to anti-Tuberculosis treatment was 90.6% within the last 4 weeks and 96.6% within the last 4 days. Multivariable analysis revealed that having treatment supporter [AOR = 3.51, 95% CI (1.15, 10.75)], difficulties in taking TB drugs regularly [AOR = 0.07, 95% CI (0.01, 0.31)], perceived benefit [AOR = 3.45, 95% CI (1.07, 11.08)] and perceived self-efficacy [AOR = 0.22, 95% CI (0.07, 0.63)] were independently associated with adherence to anti-Tuberculosis treatment.

**Conclusion:**

The treatment adherence rate of the patients was low in the last month before the data survey. Treatment supporters, difficulties in taking anti Tuberculosis drugs regularly, perceived benefit, and perceived self-efficacy were identified as affecting adherence to anti-TB treatment.

## Background

Worldwide, Tuberculosis (TB) is one of the top ten causes of death and continues to be a significant global health threat, which is the leading cause of a single infectious agent (above HIV/AIDS) [1, 2]. In 2017, TB caused an estimated 1.3 million deaths (range, 1.2–1.4 million) among HIV-negative people [1]. TB is the central public health problem in low-income countries [3, 4], and there were an additional 300,000 deaths from TB (range, 266,000–335,000) [4] among HIV-positive people [3, 5]. There were an estimated 10 million new cases of TB (range, 9.0–11.1 million), equivalent to 133 cases per 100,000 population [6, 7]. Globally, at approximately from new patients' one in five and previously treated patient one in two suffers from Multidrug-Resistant Tuberculosis (MDR-TB) [7, 8]. The World Health Organization (WHO) Global TB Report also stated that the MDR-TB rate was estimated to be 1.8% among new cases and 19% among previously treated cases in Eritrea [3]. In Ethiopia, tuberculosis is the leading cause of death and hospital admission, and the country stands 7th among the 22 highest-burden countries [9]. In Ethiopia, TB related mortality is emphasized in the top ten reported causes of death among hospital admissions, with the annual expected death rate of 26 per 100,000 populations in 2015 [10].

Adherence is frequently defined as 'the extent to which the patient's history of therapeutic drug-taking agrees with the prescribed treatment 'and suggests a contract between patient and healthcare provider [11]. Adherence to TB treatment is crucial for effective tuberculosis control that involves complex issues improving the quality of care of patients with TB [12]. The development of drug resistance is a highly man-made problem resulting from inadequate treatment due to suboptimal adherence [10]. Failure to the course of treatment or incomplete TB treatment (non-adherence) is a known cause of poor treatment outcomes, increased morbidity, depilation economy and mortality, relapse, development of drug resistance, and increased disease transmission [11, 13, 14]. The chance of developing MDR-TB among those who interrupted the treatment (for at least 1 day) [15] is higher than that of individuals who did not interrupt the treatment at all [14]. Furthermore, poverty is not only a risk factor for the development of TB, but challenges the outcomes of those on TB treatment in low-income countries [16].

Previous studies showed that non-adherence to anti-TB treatment is 50% in India, 15.5% in Thailand, 35% in Africa, 24.5%, and 19.5% in Ethiopia [15–17]. Studies show that several factors are influencing TB-treatment adherence [11–14]. Among these factors, sociodemographic characteristics’ such as age and sex [18–20], marital status [15, 21], educational level [13, 21, 22], level of knowledge about TB and its treatment [10, 18, 23], disease-related stigma [19, 24], strong social support within a patient's family and community [25–27], side effects [8] anti-TB treatment, forgetfulness' at the continuation phase, the patients' clinical symptoms subsided and felt good progress [22] are the main reasons for non-adherence in anti-tuberculosis treatment.

There are complex interactions of individual, interpersonal, socio-cultural, and health system factors that influence adherence. Understanding the level of adherence and how it relates to perception about its treatment provides an opportunity to design intervention that improves adherence through overcoming misperceptions that guide behaviour. Evidence is burgeoning on adherence and its association with socio-demographic factors. However, little is known about the relationship between adherence and patients' perceptions of TB on anti-TB treatment in terms of the health belief model. As a result, the purpose of this study was to assess non-adherence and its factors, including perceptions, using the health belief model.

## Review of health belief model constructs

The Health Belief Model (HBM) is a psychological model aimed at explaining and predicting health behaviors. The model was developed to respond to the failure of a tuberculosis screening program. The HBM theories vary between each individual and predict engagement in health-related behaviors [28–31]. The HBM distinguishes six kinds of risk perception as determinants of health behavior: perceived susceptibility, perceived severity, perceived benefits, perceived barriers, cues to action and self-efficacy [32]. Perceived susceptibility refers to an individual's perception of vulnerability to a particular risk. Individuals who feel that they are at risk of developing a particular health problem generally engage in behaviors to reduce their risk of developing it [32]. Perceived severity refers to a belief of profound effect of the condition on different aspects of life, including social and clinical consequences. Individuals who perceive a given health problem as serious are more likely to engage in behaviors to reduce its severity [32]. Perceived benefits refers to an individual's assessment of the value or efficacy of engaging in a health-promoting behavior to decrease disease risk [32]. Perceived barrier refers to a belief in the cost of undertaking an action, while patients who perceive the disease's high barrier are less adherent than those who perceive the barrier of the disease [15]. A cue or trigger is necessary for prompting engagement in health-promoting behaviors. Cues to action such as symptoms of tuberculosis, print material, pill calendars, text messages, television, radio, social media can be used to instigate compliance to tuberculosis treatment [32]. Self-Efficacy refers to an individual's perception of his or her competence to perform a behavior successfully. People are more likely to adopt healthy behavior if they think they will be successful [32].

Based on the HBM theoretical concepts discussed above, this study used the model to determine the effect of associated factors and individual patients' perceptions of TB treatment adherence.

A number of studies recommend health belief as the primary reinforcement model for improving treatment adherence, which is also cost-effective. It is, however, not widely used in Ethiopian anti-TB treatment centers and other health institutions. Previous studies of perception in tuberculosis patients in Ethiopia included both first line and second line (MDR-TB) tuberculosis patients. Previous studies did not determine health belief perceptions of the adherence outcomes of each line of treatment, and they overestimated MDR-TB patients. As a result, this study focuses solely on patients' perceptions of first-line treatment, making it more specific and allowing us to assess adherence to anti-TB treatment and associated factors among adult TB patients in Ethiopia using HBM.

## Methods

### Study design

An institution-based cross-sectional study was used to recruit study participants to determine the adherence to anti-TB treatment and associated factors among adult TB patients who were receiving their treatment at the governmental health facilities of Gondar city administration between February 20 and March 26, 2020.

### Study area and period

The study was conducted in Gondar city administration from February 20 to March 26, 2020. Gondar city administration is found in the Amhara region and is located 725 km Northwest of Addis Ababa. Gondar city administration has six sub-city and 36 kebeles (the smallest administrative unit of the Federal Democratic Republic of Ethiopia). Gondar specialized hospital and eight [8] governmental health centres provide anti-tuberculosis treatment services under Gondar city administration. The census report of 2019 found from the Gondar city health office showed that the total population was 344,046 from these 171,679 were male, and 172,367 were female. According to the health office report from compiled data of all health facilities, including Gondar specialized hospital, there were 290 TB patients within 6 months in 2019.

### Sample size and sampling procedure

The sample size was determined by the single population proportion formula (*n* = *Z*^2^*α*/2 *p* (1 − *p*) *d*2) with the assumptions of 95% confidence interval (CI) and 5% marginal error (*d*). Prevalence of adherence to anti TB treatment was taken from the previous study conducted in Addis Ababa, Ethiopia, which was 80.5% [20]. Taking 10% non-response rate the required sample size was 265.The study participants were selected using a systematic random sampling technique, and the sampling interval was two in each health facilities thus every two participants were interviewed based on their order of arrival.

### Data collection instruments and procedures

Data were collected using an interviewer-administered questionnaire. The questionnaire had five components; socio-demographic, knowledge assessment on TB and individual factors', adherence to anti-TB, health care/services and HBM constructs. A self-reporting questionnaire was used to estimate the magnitude of adherence to TB treatment among the patients in this study. Self-reporting measures are cost-effective and easy to implement. For example, a single adherence question on missed doses in the last 2 days before the interview improved results for identifying adherent patients compared to the INH (Isoniazed) urine test. It is also suggested that a measure based on trust confers accountability on the patient and enhances communication [11].

Self-reported in the last 4 days before a standardized interviewer-administered questionnaire measured survey adherence. Patients were asked to report the number of anti-tuberculosis pills they took the day before the survey and 2 days, 3 days, and 4 days before the survey. This number of pills as compared to the number of pills prescribed to the patient. The adherence to TB medication in the last 4 days was classified as Non-adherence, if 25% and more of the pills missed in the last 4 days, and recent adherence before the data collection (100%) [11, 19, 33]. Asking the patient to what extent recent tablet use in open ended questions were found to be helpful in reducing recall bias [11, 19, 33].

The patient's adherence to TB medication in the last month was also assessed using a 10 points linear VAS ("how much of your prescribed TB medications have you taken in the last month?"). Studies showed a good match between VAS, a questionnaire and INH metabolites in urine analysis. The adherence measured by the VAS was classified as Non-adherence (95%, that is rating a value lower than 95 on the VAS), and adherence (95% and above) [13, 32]. The adherence percentage was calculated as the number of doses taken by the respondent as prescribed by the clinician, divided by the number of doses prescribed to the respondent in the last 4 weeks and multiplied by 100 [11, 19, 33].

HBM constructs—Perceived susceptibility (8 items), severity (7 items), benefit and barrier [(11 and 9 items) each], cues to action (6 items) and perceived self-efficacy (6 items) having five-point Likert scale was used to assess the perception of TB patients to their adherence behaviours.

A patient whose mean score is nine or more from eleven item questions asked were considered to have good knowledge about TB. These questions were obtained from information routinely provided to patients as part of the national TB program.

One BSc nurse supervisor and six nurse data collectors were employed and trained for 2 days about the purpose of the study, timely collection of data and overall data collection procedure. Amharic version questionnaire was used to collect the data.

### Data processing and analysis

Data clean up and cross-checking were done before analysis. Data were checked, coded and entered to EPI DATA version 4.2 then it was exported to SPSS version 24 for analysis. Both descriptive and analytical statistical procedures were utilized. Descriptive statistics like percentage mean and standard deviation were used for the presentation of demographic data and prevalence of adherence. Tables were also used for data presentation. Binary logistic regression was used to identify factors associated with adherence among the TB patients. Multiple logistic regression model was fitted to control the possible effect of confounders and finally the variables which had independent association with adherence were identified on the basis of OR, with 95% CI and *p* value less than 0.05.

## Results

### Socio-demographic characteristics of respondents

A total of 265 registered TB patients were participated in this study, yielding a response rate of 100%. Their age ranged from 18 to 80 years, with a mean age of 33.34 (SD ± 10.9) years; more than half of the respondents, 146 (55.1%), were males, nearly half of participants 132 (49.8%) were married, and a majority of the participants 236 (89.1%) were Christians by their religion. One hundred nineteen of the respondents (44.9%) attended secondary and above with their educational levels, and nearly half of the participants, 125 (47.2%), had a monthly income of > 3000 Ethiopian birrs (Table 1).

**Table 1:** Socio-demographic characteristics of respondents, Gondar town city administration, Northwest Ethiopia, 2020 (*n* = 265)

	Mean
Age	33.34

		Frequency	Percent
Age	18–32	146	55.1
	33–80	119	44.9
Sex	Male	146	55.1
	Female	119	44.9
Marital status	Married	132	49.8
	Unmarried	133	50.2
Religion	Christian	236	89.1
	Muslim	29	10.9
Educational status	No formal education	92	34.7
	Primary level	54	20.4
	Secondary level and above	119	44.9
Current employment	Yes	150	56.6
	No	115	43.4
Monthly income Ethiopian birr	< 3001	140	52.8
	> 3000	125	47.2
Type of TB	PTB+	103	38.9
	PTB−	69	26.0
	EPTB	93	35.1
Type of patient	New	250	94.3
	Relapse	14	5.3
	Others * lost to follow up	1	0.4
Disclosed TB status	Yes	255	96.2
	No	10	3.8
To whom disclosed TB status	Spouse	89	33.6
	Family	166	62.6

Nearly half of the participants, 150 (56.6%), were employed in different tasks to generate incomes, while the rest did not. The majority of the participants, 250 (94.3%), were new to anti-TB treatment, followed by 14 (5.3%) relapsed TB cases. In addition, most of the participants, 103 (38.9%), were smear-positive pulmonary TB (SPPTB) cases, and 93 (35.1%) were extrapulmonary (EPTB) cases (Table 1).

### Adherence to anti-TB treatment and reasons for pill missing

**Fig. 1:**
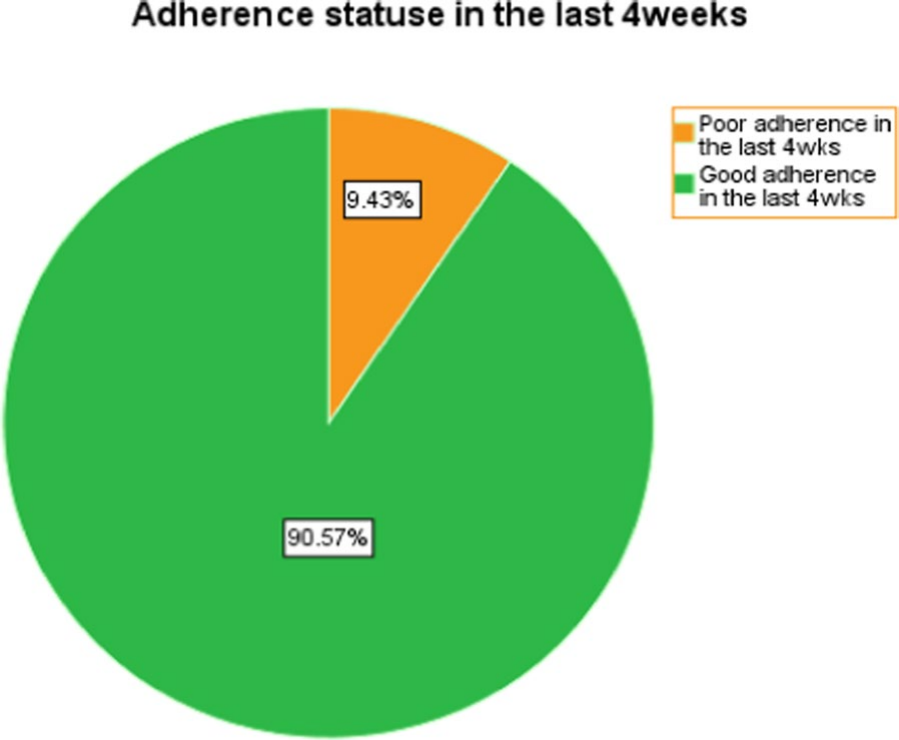
Anti-TB treatment adherence in the last 4 weeks of TB patients on anti-TB treatment in Gondar town administration, 2020 (*n* = 265)

The overall rate of adherence to anti-TB treatment was 90.6% within the last 4 weeks and 96.6% within the last 4 days (Fig. 1).

More than half of the participants, 155 (58.5%), were in the continuation phase of treatment. Nearly three-quarters of respondents, 201 (75.8%), were taking their drug with the help of someone. In addition, more than half of 154 (58.1%) were monitored to take their drug on time by their family members (treatment supporters), and 47 (17.7%) were reminded by a health professional.

**Fig. 2:**
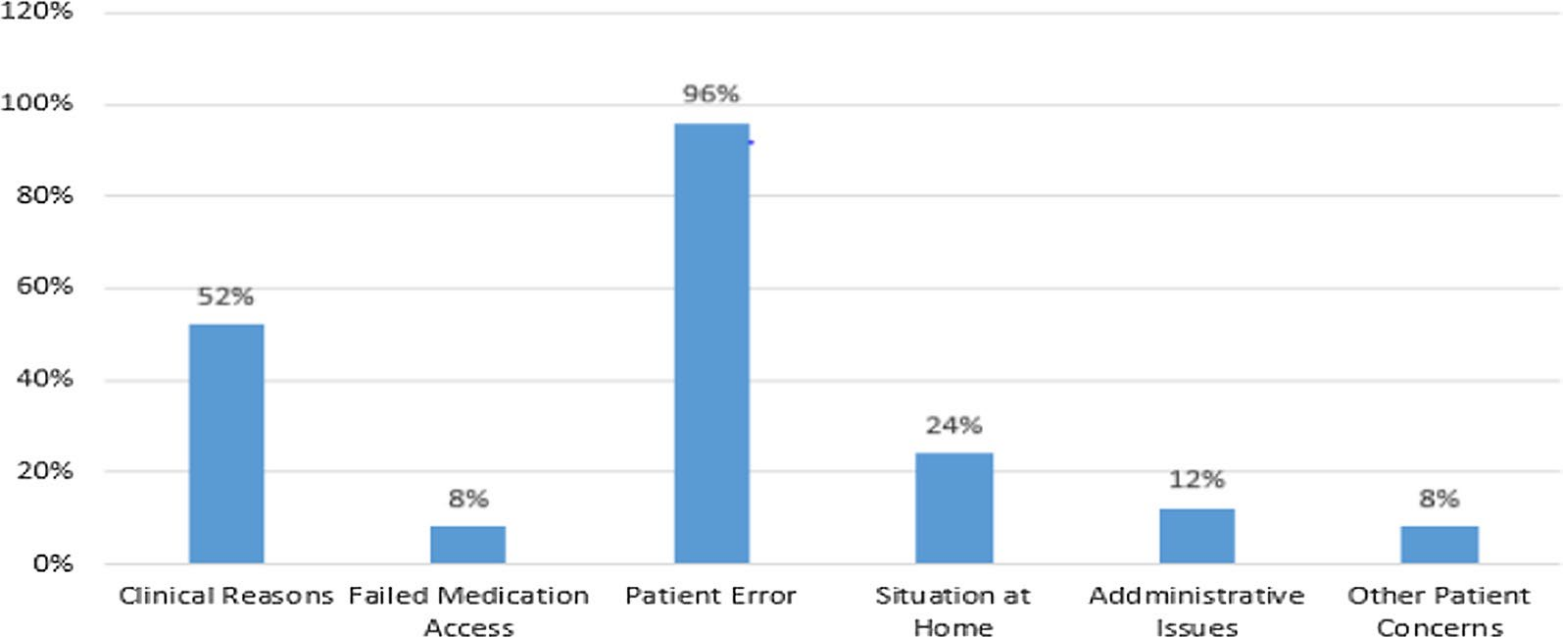
Reasons for missing medication among TB patients on anti-TB treatment in Gondar town administration, 2020 (*n* = 25)

Participants were asked about the reason for the interruption of taking medications while they report missing any number of medications. Most of the participants reported more than one reason for missing. Clinical reason (Ran out of drug supply at home, unable to get to facility for drugs /transport, unable to get to facility for drugs /sickness and drug out of stock at hospital/clinic) (52%), patient error (forgot to take the dose, felt better and so stopped taking doses, shared medications with others, lost medications, use of traditional medicines instead and had travelled and forgot drugs) (96%), situation at home (Food security problems, a family crisis, lack of support from family, lack of time due to work /other duties and depression (24%), administration issues (Clinic hours of operation not convenient, long queues/waiting time at the clinic, lack of confidentiality at clinic and discomfort with clinical staff (12%), other patients' concerns (No privacy to take medication, fear of disclosure of TB status, inadequate health care provider attitude and interference with daily issues (8%), and failed medication access (8%) were the most frequent reasons for missing the anti-TB pills (Fig. 2).

### Healthcare services and other related factors to adherence to anti-TB treatment

Most of the study participants, 180 (67.9%), lived > 2 kms from their TB treatment facility; more than half of the responds' 155 (58.5%), took an averagely of > 30 min to pick their medication. One hundred thirty-six (51.3%) used on foot as their means to pick their drugs, about half of 139 (52.5%) had to pay for transport to pick their drugs. Nearly one-third of 80 (30.2%) had any difficulties taking drugs regularly on time, and 101 (38.1%) took time off work to pick up anti-TB drugs. The majority of the participants, 255 (96.2%), disclosed their TB status to their relatives and one-third of the participants, 95 (35.8%), of those who had not disclosed it were due to fear of stigma. Two hundred and twelve 212 (80.0%) of the patients experienced adverse drug effects due to TB medicines (Table 2).

**Table 2:** Healthcare services and other related characteristics adherence anti-TB treatment of respondents', Gondar city administration, Northwest Ethiopia, 2020

		Frequency	Percent
Health education	Yes	161	60.8
	No	104	39.2
Counseling	First, visit	114	43.0
	Occasionally	96	36.2
	Never	55	20.8
Relationship with health workers	Very friendly	44	16.6
	Friendly	213	80.4
	Unfriendly	8	3.0
KM to a health facility	< 2 km	85	32.1
	> 2 km	180	67.9
Time taken HF	< 30 min	110	41.5
	> 30 min	155	58.5
Travel pick up anti-TB	On foot	136	51.3
	Public transportation	129	48.7
Take time off work to pick up your drugs	Yes	101	38.1
	No	164	61.9
Pay any transport fare to get to the health center or hospital	Yes	139	52.5
	No	126	47.5
	Total	265	100.0
Any difficulties in taking your TB drugs on time	Yes	80	30.2
	No	185	69.8
	Total	265	100.0
Felt stigmatized for having TB	Yes	95	35.8
	No	170	64.2
	Total	265	100.0
Experienced TB drugs side effect	Yes	212	80.0
	No	53	20.0

### Factors associated with adherence to anti-TB treatment

Results from logistic regression analysis, which was carried out to assess the associations of selected socio-demographic and other risk factors and the likelihood of adherence to anti-TB treatment, are summarized in Tables 3. Adherence to anti-TB treatment was considered within the last 4 weeks. In the bivariable analysis, age, educational level, treatment supporter, health education, counselling, distance from TB clinic, travelling time (single trip), type of transportation to the TB clinic, any difficulties in taking anti-TB treatment regularly, pay for transportation, perceived benefits, and perceived self-efficacy were significantly associated with anti-TB treatment adherence. However, in the adjusted analysis, treatment supporter, any difficulties in taking anti-TB treatment regularly, perceived benefits, and perceived self-efficacy were remained significantly and independently associated with anti-TB medication adherence.

**Table 3:** Bivariable and multivariable Logistic regression analysis of factors associated with adherence among patients on anti-tuberculosis treatment, Gondar city administration, Northwest Ethiopia, 2020 (*n* = 265)

Variable	Adherence status		COR 95% CI	AOR 95% CI
*Age*	Good	Poor		
18–32	137 (94%)	9 (6%)	2.36 (1.00, 5.56)	1.39 (0.49, 3.92)
33–80	103 (87%)	16 (13%)	1	
*Educational level*				
No formal education	84 (91%)	8 (9%)	1	
Primary level	45 (82%)	9 (16%)	0.47 (0.17, 1.31)	0.35 (0.10, 1.21)
Secondary level and above	111 (93%)	8 (7%)	1.32 (0.47, 3.66)	1.06 (0.30, 3.80)
*Employment*				
Yes	132 (88%)	18 (12%)	0.47 (0.19, 1.18)	0.54 (0.18, 1.63)
No	108 (94%)	7 (6%)	1	
*Treatment supporter*				
Yes	186 (93%)	15 (7%)	2.29 (0.97, 5.40)	3.51 (1.15,10.75)**
No	54 (84%)	10 (16%)	1	
*Health education on anti TB*				
Yes	149 (93%)	12 (7%)	1.77 (0.77, 4.05)	0.85 (0.26, 2.71)
No	91 (87%)	13 (13%)	1	
*Counseling*				
First	104 (91%)	10 (9%)	1.77 (0.65, 4.77)	1.54 (0.39, 6.13)
occasionally	89 (93%)	7 (7%)	2.16 (0.73, 6.33)	1.81 (0.38, 8.43)
Never	47 (85%)	8 (15%)	1	
*Distance from TB clinic (single trip) (km)*				
< 2 km	81 (95%)	4 (5%)	1	
> 2 km	159 (88%)	21 (12%)	0.37 (0.12, 1.12)	1.13 (0.14, 8.97)
*Traveling time (single trip) (min)*				
≤ 30	105 (95%)	5 (5%)	3.11 (1.13, 8.56)	3.03 (0.48, 18.87)
> 30	135 (87%)	20 (13%)	1	
*Type of transportation to the TB clinic*				
walking/foot	126 (93%)	10 (7%)	1	
Public transport	114 (88%)	15 (12%)	0.60 (0.26, 1.39)	1.15 (0.21, 6.27)
*Pay for transportation*				
Yes	123 (88%)	16 (12%)	0.59 (0.25, 1.39)	0.38 (0.06, 2.34)
No	117 (93%)	9 (7%)	1	
*Any difficulties in taking TB drugs regularly*				
Yes	67 (84%)	13 (16%)	0.35 (0.15, 0.82)	0.14 (0.04, 0.42)**
No	173 (94%)	12 (6%)	1	
*Perceived susceptibility*				
High	118 (92%)	10 (8%)	1.45 (0.62, 3.35)	
Low	122 (89%)	15 (11%)	1	
*Perceived severity*				
High	109 (91%)	11 (9%)	0.94 (0.41, 2.16)	
Low	131 (90%)	14 (10%)	1	
*Perceived barrier*				
High	102 (89%)	13 (11%)	0.68 (0.29, 1.55)	
Low	138 (92%)	12 (8%)	1	
*Cues to action*				
High	106 (91%)	11 (9%)	1.00 (0.43, 2.30)	
Low	134 (91%)	14 (9%)	1	
*Perceived benefits*				
High	95 (94%)	6 (6%)	2.07 (0.79, 5.38)	3.45 (1.07, 11.08)**
Low	145 (88%)	19 (12%)	1	
*Perceived self-efficacy*				
High	73 (83%)	15 (17%)	0.29 (0.12, 0.67)	0.22 (0.07, 0.63)**
Low	167 (88%)	10 (12%)	1	

**Variables that were found to have significant association during multivariable analysis at *p* value < 0.05

## Discussion

The objective of this study was to determine the extent of adherence to anti-TB treatment and identify factors that influence TB treatment adherence among persons on anti-TB treatment for at least 1 month in Gondar town administration. Multivariable logistic regression analysis revealed that treatment supporters, any difficulties taking anti-TB drugs regularly, perceived benefit, and perceived self-efficacy could independently predict TB treatment adherence.

This study showed that overall self-reported adherence in the last 1 month before the survey were 90.6% (95% CI = 86.6–94.3) in Gondar city. This finding was in line with the studies conducted in northwest Ethiopia (90%) and northeast Ethiopia (88.5%) [11, 22]. However, this finding is lower compared to studies done in the Republic of Tanzania (95.7%) and Northern Ethiopia (97.3%) (8, 25). The possible explanation for the variation in adherence was may be due to the data collection time being in the Lent fasting period. As a result, and as it is customary for most Ethiopian Orthodox church Christian members to go to holy water sites for a week or a fortnight for a healing purpose, they miss their medication. Also, in Tanzania, a direct measurement of metabolites' presence/concentration of adherence to anti-TB by urine test and confirming whether isoniazid has been taken in the last 24–30 h were used.

However, this study reported a higher adherence rate compared to study findings from India (84.50%), Uganda (65%), Equatorial Guinea (78.57%), Addis Ababa, Ethiopia (80.5%), and Southern Ethiopia (74%), (15, 17, 26, 32, 33). This difference in the adherence level was probably due to differences in adherence measurement tools and sampling techniques. Southern Ethiopia's study used multi-stage stratified sampling techniques with Morisky adherence scales tools (MAS) measurements. Equatorial Guinea used Morisky–Green–Levine test tools. Study populations of both studies [17, 26] were rural and urban mixed populations. However, our study population was urban dwellers.

The current study revealed that patients with treatment supporters were highly likely to be more adherent than those who had not treatment supporters. This was justified because patients were supported by friends and families to reduce psychological stress and financial burden, especially in our environment where the extended family system is practised. This finding is supported by existing literature on treatment supporters' family members, friends, and other volunteers to enhance adherence by emotional and financial support. Many patients reported that they were forced to seek support due to loss of income and physical tiredness. The respondents underscored that any support, especially from family members, was an essential factor in continuing their treatment. This was in line with a study done by north-central Nigeria, Eritrea, Guinea, and Addis Ababa, Ethiopia [5, 19, 28, 34].

The current study showed that those patients who had so many operational problems during the treatment in taking TB drugs regularly were less likely to be non-adherent. This could be because our daily activities have many challenges to survive and generate daily incomes, which could affect the time to take TB drugs regularly, like an unfitted schedule of working time, daily labour, distance from the work area, and loading work. This finding is consistent with other studies done in Andhra Pradesh, India, Addis Ababa, Ethiopia [15, 30].

This study revealed that HBM constructs perceived benefits were significantly associated with adherence to anti-TB treatment. These results supported the idea that HBM constructs might contribute to predicting therapeutic adherence in TB patients. Furthermore, the perceived benefit was the critical factor influencing adherence, meaning participants who understood the benefit of treatment were more adherent. The current study also showed that patients with high perceived benefits were 3.4 times more likely to adhere than those with low perceived benefits. This finding is consistent with other studies done in Irian and Addis Ababa, Ethiopia [21, 29].

Self-efficacy increases as the patient successfully resist temptation in high-risk situations. However, the study showed that TB patients with high perceived self-efficacy were less likely to be adherent to anti-TB treatment, while this finding contradicted other studies. This could be explained; high self-efficacy negatively affected one's motivation to do something that the person already adopt. One manifestation of high self-efficacy is the tendency to overestimate one standing on a dimension of judgment or performance and lack of trust in the diagnoses with treatments. Patients with high perception towards anti-TB treatments at continuation phases felt well-being due to the subsiding of more clinical signs and symptoms. Similarly, the previous study revealed that the main reason TB patients stop taking their treatment before they are cured is that 54% of the respondents feel this is caused by "patients feel better and think they are cured [11, 24, 35]. Finally, designing qualitative research to know more reasons for contradicted results in self-efficacy are warranted.

## Limitation

This study shares the limitations of cross-sectional studies, and after this, it might suffer from temporal relationship establishment with some variables and might not provide much more substantial evidence of causality. In addition to this, as the study is only addressed, a self-reported recall bias might lead to an under-estimated finding. On the other hand, as health care workers at the facilities collected the data, the result might be under-reported by the social desirability bias. HBM has some limitations. For example, the HBM does not account for other factors that may prevent an individual from practising the desired behaviours, such as environmental factors.

## Conclusion

The treatment adherence rate of the patients was low in the last month before the data survey, and there was an improvement in the treatment levels compared with previous studies. This study identified factors such as treatment supporter, any difficulties in taking anti-TB drugs regularly, perceived benefit, and perceived self-efficacy as affecting adherence to anti-TB treatment. Moreover, the results of our study indicate that HBM is a suitable model in predicting therapeutic adherence in TB patients. Therefore, to plan effective TB prevention interventions, health care workers should target programs to minimize perceived barriers and promote perceived benefits, improved adherence to anti-TB treatment. Besides, our results highlight the importance of perceived benefit in TB treatment adherence, which health educators should consider when developing programs on motivation for treatment adherence.

### Footnotes

^a^Keble is the smallest administrative unit of the Federal Democratic Republic of Ethiopia.

## Data Availability

All data and material related to this article will be made available upon request.

## Abbreviations

BI: Behavioral intention
CPR: Contraceptive prevalence rate
EDHS: Ethiopian demographic health survey
FP: Family planning
HC: Health center
IUD: Intra uterine device
LAPMs: Long acting and permanent contraceptive methods
LACMs: Long acting contraceptive methods
MoH: Ministry of health
PBC: Perceived behavioral control
RH: Reproductive health
SPSS: Statistical package for social sciences
SSA: Sub- Saharan Africa
TPB: Theory of planned behavior
WHO: World health organization
